# Ferrites with a Minimized Secondary Electron Yield

**DOI:** 10.1002/advs.202410083

**Published:** 2025-01-17

**Authors:** Robin Uren, Manuel Hoffman, Amin Din, Stefan Wackerow, Holger Neupert, Stephan Pfeiffer, Alice Moros, Michael Barnes, Giorgia Favia, Marcel Himmerlich, Amin Abdolvand

**Affiliations:** ^1^ School of Science and Engineering University of Dundee Nethergate Dundee DD1 4HN UK; ^2^ CERN European Organisation for Nuclear Research Geneva 23 Geneva 1211 Switzerland

**Keywords:** ferrites, laser materials processing, secondary electron yield

## Abstract

Ferrites are an essential material in modern industry due to their exceptional magnetic properties and high resistivity. Many applications of ferrites necessitate exposure to high energy electrons, particularly space science and particle accelerators, where charging, multipacting, and electron clouds (ECs) are major issues. ECs are of particular concern around the Ni/Zn soft ferrite kicker magnets as the large hadron collider (LHC) undergoes its high luminosity upgrade. Here, laser engineered surface structures (LESS) are used to reduce the secondary electron yield (SEY) of Ni/Zn ferrites from 2.1 to its experimentally determined minimum of 0.81, eliminating the ferrites ability to contribute to the formation of ECs. This demonstrates the applicability of LESS outside of metals and its broader applicability to reducing the SEY of technical materials.

## Graphical abstract

Ferrites are a key material in particle accelerators and space science, exposing them to high energy electrons and leading to multipacting, charging and electron cloud formation. Laser engineered surface structures are shown to dramatically reduce the secondary electron yield of ferrite eliminating these issues. This is also the first investigation into ferrite optical and light matter interactions.

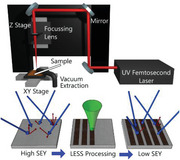

## 1 Introduction

Ferrites are the preferred material in high frequency transformer and electronic components as well as microwave absorbers due to their low coercivity, leading to minimal hysteresis losses, high resistivity, minimizing eddy currents, and low cost.^[^ ^1^, ^2^, ^3^, ^4^ ^]^ Ferrite's vacuum compatibility also makes it advantageous for use in space science radio frequency (RF) equipment^[^ ^5^, ^6^ ^]^ and as the yoke of kicker magnets in particle accelerators, where it allows the magnet to be closer to the beam, increasing force transfer.^[^ ^7^, ^8^, ^9^, ^10^ ^]^ These latter applications necessitate exposure to high energy electrons. These, primary electrons (PEs), will frequently eject electrons, known as secondary electrons (SEs), from the lattice. The ratio of SEs to PEs is called the secondary electron yield (SEY), see Equation (1).

(1) $$\;\delta \left(E\right)=\frac{N_{e}}{N_{i}}$$

δ(*E*) is the SEY at a given primary electron energy (PEE), *E*, *N*_*e*_ is the number of emitted electrons and *N*_*i*_ is the number of electrons incident. Most materials have an SEY greater than one which leads to problems in vacuum as there is no ground connection to replenish the electrons nor a material to absorb escaped electrons. For satellites this leads to charging and multipactor failure while for particle accelerators the major issue is the generation of ECs.

In an accelerator, SEs can gain energy from the particle beam and re‐impact the surface causing a positive feedback loop. These SEs build into said ECs.^[^ ^11^, ^12^ ^]^ This is a common issue at the Large Hadron Collider (LHC) and Super Proton Synchrotron (SPS) at CERN where ECs have been observed in several areas including the interior of the beam screens, near the compact muon solenoid experiment and at several different kicker magnets limiting performance.^[^ ^11^, ^13^, ^14^, ^15^ ^]^ ECs then lead to vacuum degradation, beam instabilities and losses, and heating within the beam chamber.^[^ ^14^, ^16^, ^17^, ^18^, ^19^ ^]^ Resolving this issue requires an innovative approach that recognizes the engineering constraints of operating within a particle accelerator.

This paper focuses on mitigating ECs occurring around ferrite kicker magnets. At CERN two specific ferrite compositions are currently used, 8C11 from Ferroxcube^[^ ^20^ ^]^ and CMD 5005 supplied by National Magnetics Group Inc.^[^ ^21^ ^]^ These are both Ni/Zn soft ferrites and produce magnetic fields that can track a current rise to within 1 ns, have a low remnant field, and minimal out‐gassing after bake out, making them ideal for fast switching magnets operating in vacuum.^[^ ^10^, ^22^ ^]^ This material is directly exposed to the particle beam, making its SEY a contributing factor to ECs in the particle accelerators. The upcoming high‐luminosity upgrade for the LHC at CERN exacerbates this issues due to the increased bunch population.^[^ ^18^ ^]^ The kicker magnets are responsible for the injection and extraction of particle bunches to the beamline of an accelerator. An LHC‐type beam batch is injected or extracted during one kicker magnet pulse. Maximizing the collision signal of a particle accelerator requires minimal batch spacing which is limited by the kicker magnet rise and fall time. Currently, magnetic rise times of kicker magnets are sub‐microsecond in the LHC, and as fast as 115 ns in the SPS.^[^ ^7^, ^8^, ^23^, ^24^ ^]^ ECs are a particular concern as they, along with beam coupling impedance, drive heating of the ferrite yoke.^[^ ^10^ ^]^ Any SEY reduction of the ferrite cannot impede the magnets field strength, field rise time, greatly increase impedance or compromise vacuum compatibility for it to remain viable as a kicker magent material.

Continuous low SEY metallic shielding between the beam and the ferrite is not a feasible solution, as it is in other areas,^[^ ^25^, ^26^ ^]^ as eddy currents induced by the rapid changes of the kicker magnetic field degrade the field rise and fall times. Other mitigating factors, including impedance reducing screen conductors or conducting fingers applied parallel to the beam have been investigated.^[^ ^15^, ^27^, ^28^ ^]^ However, these solutions only cover a relatively small surface area and the overall SEY of the ferrite still needs to be reduced. Limited research has been devoted to the reduction of SEY in dielectrics and semiconductors, as often another low SEY material can be used to shield them from electrons. SEY reduction in ceramics has been primarily concerned with increasing the flashover voltage (which is linked to SEY) through low SEY coatings. There is a particular focus on RF alumina windows suffering from multipactor breakdown,^[^ ^29^, ^30^, ^31^, ^32^, ^33^, ^34^, ^35^ ^]^ including those at the CERN and KEKB accelerators.^[^ ^36^, ^37^, ^38^ ^]^ Coating technologies are relatively cheap to implement after investing in the deposition machine and have been proven robust in high energy vacuum environments. The two main coatings used are tin nitride (TiN) and various forms of carbon coatings. These coatings, especially TiN rarely reduce the SEY below one.^[^ ^38^, ^39^, ^40^, ^41^ ^]^ These are typically not applicable to ferrite kicker magnets as they have a high surface conductivity, leading to eddy currents and magnetic field degradation.^[^ ^42^, ^43^, ^44^, ^45^, ^46^ ^]^

**Figure 1:**
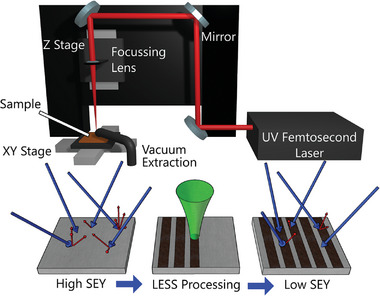
Illustration of the experimental setup and basic methodology for SEY reduction with LESS.

Recently, we have devised a new, highly effective, method of reducing the SEY of metal surfaces through implementing laser engineered surface structures (LESS).^[^ ^13^, ^47^, ^48^, ^49^, ^50^, ^51^, ^52^ ^]^ LESS treatment uses a pulsed laser to scribe micro‐scale trenches into the surface and simultaneously produce a fine nanostructure. An illustration of the simple setup and methodology is given in **Figure** **1** with further details in Section Laser and Motion Control Setup. These nested, high aspect ratio trenches greatly increase the chance of any given SE to re‐impact the surface and be absorbed.^[^ ^53^, ^54^, ^55^ ^]^ This topographical effect is independent of the bulk properties of the material, implying the effectiveness of this method on non‐metals. The process also induces changes to the surface composition which can also alter the overall SEY. For example, when implementing LESS to copper while using nitrogen as a displacement gas, an increase in the prevalence *Cu*_2_ *O* was found, which has a lower SEY than air exposed *Cu*, contributing to the measured reduction in SEY.^[^ ^48^, ^52^, ^56^ ^]^ This effect is material dependant but generally less impactful than the topographical effects as it cannot reduce SEY below that of the newly present materials. The technique can be similarly cost efficient to coatings after the investment of the laser system but may have a longer processing time. However, this is mitigated by the ability to selectively process areas tackling only the regions where the majority of PEs impact. It is also capable of achieving much lower SEYs below one and allows greater flexibility in tailoring a balance between SEY reduction and changes in other surface properties such as beam coupling impedance or conductivity.

Dielectric and semiconducting materials under intense ultrafast laser irradiation generate large numbers of free electrons,^[^ ^57^, ^58^, ^59^ ^]^ allowing the material to follow a similar ablation pathway as metals. This opens the possibility of performing LESS processing on non‐metals. Employing ultrafast (sub picosecond) laser pulses enables more precise structuring, wavelength insensitivity and a reduced heat affected zone. This confines material modification and removal to a small surface layer reducing the likelihood of negatively impacting bulk properties of the material, for example mechanical strength or magnetic properties.^[^ ^50^, ^60^ ^]^

Here, LESS processing has been applied to the dielectric ceramics (Ni/Zn ferrites), used in the kicker magnets at CERN. This is the first time that this technology is applied to a non‐metal, enabled by the application of ultrafast UV laser pulses. Before processing could occur the optical and light matter behaviour needed to be characterized as Ni/Zn Ferrite has never previously been studied under ultrafast ablation, and there has been limited study of its SEY.^[^ ^10^, ^33^ ^]^ Here we present, for the first time, the wide spectrum optical and light matter behaviour of this crucial material, along with the successful reduction of its SEY.

## 2 Results and Discussion

### 2.1 Surface Properties of Ferrites

To optimally laser process Ni/Zn ferrites, first one must understand their surface properties and behaviour under high intensity optical, and low energy electron bombardment. All experiments are carried out on samples with dimensions of 10 × 10 × 2 mm. As ferrites are semiconductors, laser pulses will first excite electrons from the valence to the conduction band before ablation occurs.^[^ ^61^ ^]^ Therefore, ablation will be more efficient for photon energies greater than the bandgap as single photon excitation to the conduction band becomes possible. Once the conduction band is heavily populated, the material will have metal like optical properties, leading to similar SEY reducing micro‐scale surface features. One key difference will be the nanostructure, which is largely formed by material re‐solidifying on the surface. Ferrite's higher melting point,^[^ ^62^ ^]^ and compound nature will lead to different surface structures and more complex changes to the surface composition than with metals.

An analysis of the elemental composition of the top few microns of ferrite samples' surfaces was conducted by energy dispersive X‐ray spectroscopy (EDX). The chemical composition is displayed in **Table** **1**. The carbon is thought to originate largely from hydrocarbon contaminants on the surface. We observe approximately 50% more zinc than nickel. This ratio of dopants indicates a bandgap of approximately 1.59 eV.^[^ ^63^ ^]^ This indicates more efficient ablation will be achieved for wavelengths below 780 nm.

**Table 1:** The surface composition of our untreated ferrite samples, in atomic percentage (at.%) as determined by EDX. The “Other” column denotes the sum of at.% of all other trace elements detected, largely aluminium and silicon.

	C	Fe	O	Zn	Ni	Other
8C11	25.6	19.7	41.5	5.5	3.5	4.2
CMD 5005	19.4	25.6	42.9	7.3	4.6	0.2

**Figure 2:**
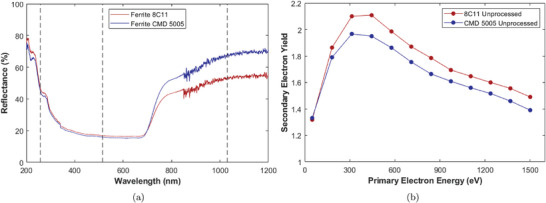
Surface properties of untreated Ni/Zn ferrite. The left graph shows the materials reflectivity corrected against an alumina baseline sample. Black dashed lines indicate available laser processing wavelengths. The right shows the SEY of unprocessed 8C11 and CMD 5005 ferrites. Each SEY data point has a statistical error of ±0.05.

To understand ferrites' interaction with different lasing wavelengths it is important to understand its bulk reflectivity which has received limited study.^[^ ^63^, ^64^ ^]^ Both samples reflectivity between 200 and 1200 nm is plotted in **Figure** **2**. Absorption was also measured although neither sample showed any transmission in this range, indicating the absorption depth is shorter than 2 mm for all wavelengths tested. Both samples have similar reflectivity, although the CMD 5005 is more reflective through the near infra‐red. We see the lowest reflection through the visible range, i.e., around the band‐gap energy.

The ferrite sample's SEY was measured prior to laser processing. The SEY is measured by monitoring the sample current, *I*_*s*_, and the secondary electron current as measured by a positively biased collector, *I*_*c*_. As ferrite is an insulator a pulsed measurement technique is used to ensure accurate measurement. These signals are numerically integrated across the pulse to give the total charge collected by the collector, *Q*_*c*_ and on the sample *Q*_*s*_. The SEY is then determined according to Equation (2). Full details in Section SEY Measurement. Looking to the SEY curves plotted in Figure 2b, it is found that our results are consistent with previous measurements.^[^ ^23^, ^65^ ^]^ The 8C11 ferrite has a maximum SEY, δ_*max*_, of 2.1 at a PEE of ∼450 eV, with the CMD 5005 being slightly lower at 1.95 with a PEE of ∼320 eV. We believe the CMD 5005's slightly lower δ_*max*_ is due to it being isostatically pressed at the end of the manufacturing process. This results in a slightly higher density of 5.270 gcm^−3^ compared to 5.100 gcm^−3^ for 8C11. Denser materials typically have shorter electron mean free paths, reducing the chance electrons will escape the material before losing all their energy and therefore have a lower SEY.^[^ ^66^, ^67^ ^]^ It was also found that CMD 5005 has a slightly lower average surface roughness of 1.75 µm compared to 8C11 at 2.53 µm. This was measured according to the parameters given in Section Surface Roughness Measurements. This decreased roughness is likely also the result of the isostatic pressing. It also helps explain the higher SEY of 8C11 as higher surface roughness at low aspect ratios means PEs impact at higher angles meaning SEs are generated nearer the surface and are more likely to escape, raising the SEY.

(2) $$\delta =\frac{Q_{c}}{Q_{c}+Q_{s}}$$

Achieving metal‐like ablation pathways when laser processing a dielectric requires operating within the two temperature regime and therefore the use of sub picosecond laser pulses.^[^ ^59^, ^68^, ^69^ ^]^ In this regime electrons within the illuminated region undergo rapid thermalization, while the lattice remains cold as this energy can only be transferred via electron ‐ phonon transitions that occur on the timescale of multiple picoseconds. Material removal therefore occurs via ablation with limited energy transfer to the lattice, reducing heating compared to continuous wave or longer pulse laser processing. All experiments within this paper are therefore conducted using 200 fs pulses with a 400 kHz repetition rate. Ferrites' higher absorption at 515 nm would make it more efficient for material removal if used in continuous wave or longer pulse processing. This will not necessarily correlate with the multiphoton absorption from 200 fs pulses, and may instead lead to more unwanted heating.

To derive effective processing parameters, first the damage threshold (DT) of the ferrite must be measured. This is done for 1, 10, 100, and 1000 pulses per spot (PPS) using the method outlined by Kirkwood et. al.^[^ ^70^ ^]^ The diameter of damage spots is measured as the peak fluence of the pulses is increased. The relative diameter squared, *D*_*Rsq*_, given as $D_{Rsq}=\frac{D^{2}}{w_{0}^{2}}$, where *D* is the measured damage diameter and *w*_0_ is the beam diameter, is then plotted according to Equation (3). Here ϕ_*pk*_ is the peak fluence and ϕ_*th*_ is the minimum laser fluence required for ablation of the material. This method is insensitive to focal spot size which allows us to achieve similar maximum fluences across all three wavelengths. Subsequently, the 1030 nm is focused to a 59 µm diameter, the 515 nm to 33 µm and the 257 nm 20 µm. This gives maximum available fluences of 7.3, 11.7, and 6.4 J cm^−2^ respectively.

(3) $$D_{Rsq}=\frac{1}{2}ln\left(\frac{\phi_{pk}}{\phi_{th}}\right)$$

**Table** **2** summarizes the DTs of the samples, showing a clear damage incubation effect. No samples showed any visible damage (as seen under an optical microscope) at one PPS, damage was only observed at 10 PPS in the infrared whilst all wavelengths produced damage with 100 PPS. Damage incubation in ceramics can occur either from various forms of non‐thermal, nanoscale damage, such as defect creation, or from thermal accumulation.^[^ ^3^, ^71^, ^72^, ^73^ ^]^ We suspect non thermal damage to be the dominant mechanism for 515 nm and 257 nm as at threshold damage there is no observable signs of melt flows. This is corroborated by the lack of further damage incubation as we move from 100 to 1000 pulses per spot, implying that initial nanoscale damage lowers the DT to where large scale ablation can occur. For 1030 nm it is less clear which mechanism is dominant.

**Table 2:** DTs, in J cm^−2^ of the ferrite samples tested at three wavelengths for 10, 100, and 1000 PPS. No sample showed visible damage at 1 PPS.

Laser Parameters		Damage Threshold (J cm^−2^)		
Wavelength (nm)		10 PPS	100 PPS	1000 PPS
8C11	257 nm	—	0.14 ± 0.08	0.10 ± 0.04
	515 nm	—	0.40 ± 0.05	0.39 ± 0.05
	1030 nm	0.20 ± 0.14	0.10 ± 0.08	0.09 ± 0.03
CMD	257 nm	—	0.12 ± 0.12	0.05 ± 0.06
	515 nm	—	0.40 ± 0.01	0.39 ± 0.09
	1030 nm	0.50 ± 0.26	0.12 ± 0.13	0.12 ± 0.09

**Figure 3:**
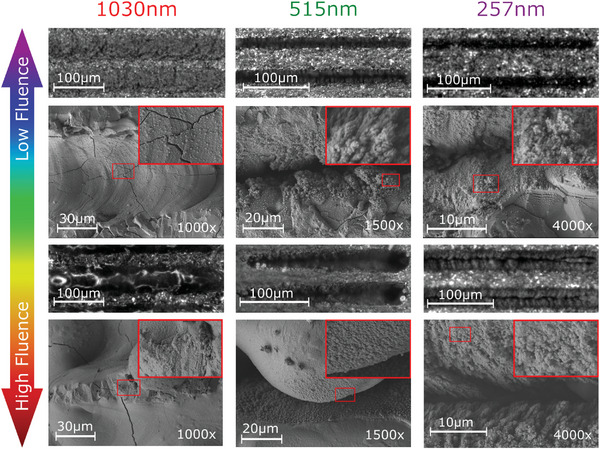
Optical (top and third row) and SEM images (second and bottom row) of the ferrites (all 8C11) after DT testing with 1000 pulses per spot. Top rows show images near the threshold fluence. While the bottom rows are at many times the DT, the SEM images are at specifically 3x, 5x, and 30x the DT for 1030, 515, and 257 nm wavelengths, respectively. Red inserts show nanostructure detail.

As expected, multiphoton absorption was more significant to the DT of ferrites than single photon. This confirms ablation as the major damage mechanism at lower fluences. **Figure** **3** shows selected images captured using a digital optical microscope of the 1000 PPS DT test trenches. These are alongside scanning electron microscope (SEM) images detailing the surface features and nanostructure. Clear differences can be seen in the light matter interactions for the different wavelengths. We see V shaped trenches with a feathered nanostructure for both 257 and 515 nm irradiation near the DT. At higher DTs the 257 nm trenches remained consistent while 515 nm exhibited significant melt flows, caused as heat built up during processing. This starts at approximately 5 times the DT and is exacerbated as the fluence increases. The feathered nanostructure that remains after ablation is well designed for electron trapping due to its high aspect ratio features. In contrast, the rounded bumps found on the smooth melt flows are an ineffective nanostructure for SEY reduction. The consistency in light matter interaction at 257 nm makes it much more attractive for laser processing ferrites as it will lead to more consistent results.

In contrast, 1030 nm irradiation led to U shaped trenches with a smooth reflective surface broken by cracks formed from rapid cooling. These are covered in a similar low‐density nanostructure as seen between the trenches. This is formed from material re‐solidifying out of the plasma plume formed above the surface during processing. Significant melt flows occur at higher powers disrupting the trench shape. This shows a strong thermal pathway at this wavelength, implying more material removal from thermal melt and vaporization, similar to results seen in alumina.^[^ ^74^ ^]^ Smooth glass like surfaces are to be avoided as glasses tend to have SEYs greater than 2.^[^ ^75^, ^76^, ^77^ ^]^

It's thought that the 257 nm wavelength is providing these advantageous structures for SEY reduction due to its higher photon energy of 4.8 eV. The work function for these materials is unknown, however, there are limited reports for similar materials giving the work function on the order of 5 eV.^[^ ^78^, ^79^ ^]^ This means two photon ablation is the major source of material removal and possibly even single photon ablation after the material has been excited by previous pulses. The longer wavelengths require more photons to fully remove an electron, leading to a greater statistical chance that an excited electron will remain in the material and redeposit the energy as heat, leading to melting. This can degrade nanostructure and fill in micro scale trench features reducing the occurrence of high aspect ration features needed for SEY reduction. Femtosecond laser ablation itself is also the likely source of the nanostructure though the exact mechanism is hard to identify.^[^ ^80^ ^]^

### 2.2 LESS Processing for Reduced SEY

From the observations in Section 2.1, it is clear that 257 nm is the optimal wavelength for SEY reduction in ferrites. Initial test processing, designated “Alpha,” targeted low SEY and was performed on both 8C11 and CMD 5005 Ni/Zn ferrite. The focal spot size was kept small, at a 25 µm diameter, to ensure high aspect ratio trenches whilst maintaining an acceptable processing speed of 10.4 mm s^−1^. The average power was set to roughly double the DT at 125 mW (giving a 0.13 J cm^−2^ peak fluence) to ensure significant headroom for ablation whilst moderating trench depth to minimise effects on other material properties such as resistivity, beam coupling impedance and/or magnetic permeability. The hatch distance (distance between trenches) was set at 20 µm and the surface was impacted by 1000 pulses per spot.

**Figure 4:**
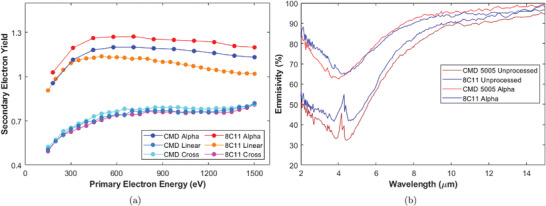
a) The SEY of the ferrite samples processed with Alpha, Linear and Cross parameters. See Section 2.2 and Table 3. Note the floor in SEY of δ_*max*_ 0.81 found with the 8C11 Cross and CMD 5005 Linear and Cross parameters. Each data point has an approximate error in the SEY of ±0.05. b) The infra red, optical emissivity of the Alpha series and unprocessed ferrite, details of measurements given in Section Optical Spectroscopy.

One sample of each ferrite was laser processed with these parameters. Post‐processing, and with no further modifications, the SEY was dramatically reduced to a maximum of 1.27 at a ∼700 eV PEE and 1.2 at a ∼575 eV PEE for 8C11 and CMD 5005 ferrite respectively (see SEY curves in **Figure** **4**a). The lower SEY of the CMD Alpha sample is attributed to its higher density leading to its lower SEY before processing. Our methodology has been highly effective at lowering SEY in a previously under‐investigated material, demonstrating it can be applied broadly in order to lower the SEY of surfaces.

An additional benefit was found in a notable increase in emissivity of the samples, see Figure 4b. The samples post processing have a dark black colour and measurements show an increased emissivity across the measured spectrum from 2 to 14 µm, particularly at the shorter wavelengths. This is beneficial as it will help ferrite components to remove any accumulated heat which can be a significant issue in both kicker magnets and space  science.

### 2.3 Improved LESS Processing for Minimal SEY

Whilst this is a significant reduction, an SEY above unity can still lead to electron cloud formation. Observations under optical and scanning electron microscopes revealed the trenches to be ∼14µm wide with a convex V shaped profile. This is significantly narrower than the beam diameter of 26 µm. This is explained by the gaussian profile of the beam with 7µm corresponding to the radius at which the localized intensity drops to one half, i.e. below the DT. The trench coverage, and therefore electron trapping potential of the surface could therefore be improved by narrowing the hatch distance and increasing trench density. Creating higher aspect ratio trenches will also produce a lower SEY. Achievable through increasing the total laser fluence via reducing the laser spot size, increasing the average laser power, increasing the number of pulses per spot or a combination of all three. A set of parameters described in **Table** **3** was therefore created to explore these possibilities and further reduce the SEY.

**Table 3:** Laser parameters used for the ferrite samples with improved SEY reduction. The spot diameter was reduced to 20 µm and the hatch distance reduced to 15 µm. The scan speed is therefore reduced to 7.6 mm s^−1^ giving 1053 PPS.

Sample and	8C11	8C11	CMD	CMD
Trench Pattern	Linear	Cross	Linear	Cross
Average Power (mW)	125	125	250	250
Pulse Energy (µ J)	0.32	0.32	0.63	0.63
Fluence (J/cm^2^)	0.20	0.20	0.40	0.40
DT Multiple	∼3	∼3	∼6	∼6

To target the lowest possible SEY the focal spot size was reduced for all samples, increasing the fluence. For the CMD samples the average power was also increased. These adjustments target deeper, higher aspect ratio trenches. The hatch distance is reduced to 15 µm minimizing the surface area not ablated by the laser. The pulses per spot were roughly maintained in order to not excessively increase processing time. For each sample a cross hatched laser pattern was made with the same parameters, further increasing trench density.

**Figure 5:**
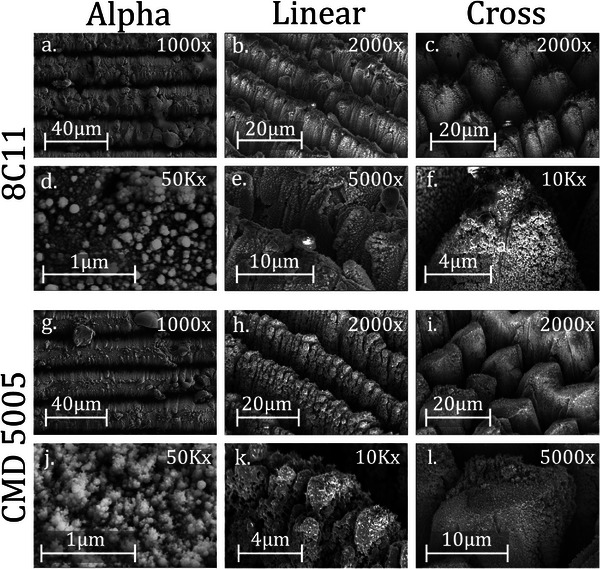
SEM images of the processed ferrite samples. Alpha images are taken from directly above, others from a 50 ° angle. The top row for each form of ferrite shows the overall structure of each processed sample. The second row shows details of the nanostruture. The nanostructure for 8C11 Alpha is taken between the trenches and within the trenches for CMD 5005 Alpha. Note the very different structures created when the ablation threshold is reached.

These new processing parameters have further reduced the SEY, see Figure 4a. The 8C11 Linear parameters reduced the SEY to a maximum of 1.14 at ∼500 eV PEE. Increasing the trench density with a cross pattern has the desired effect of dropping δ_*max*_ to 0.81. The shape of the curve is also dramatically changed with greater suppression at low PEEs rising to a peak at the highest energy measured, 1500 eV. The SEY curves for both CMD samples are almost identical to that of the 8C11 Cross sample with a peak of 0.81 at a 1500 eV PEE. This clear floor in the SEY demonstrates we have found the minimum SEY attainable for ferrites with LESS. This minimum is hypothesised to exist because beyond a certain depth within a “V” shaped trench, near total electron absorption is achieved. Further deepening of the trenches therefore yields no further reduction in SEY. We have also nearly fully coated the surface in a nanostructure with strong electron absorption, see **Figure** **5**f,k. The SEY is therefore set by the properties of the nanostructure and surface chemistry of the upper areas of these trenches. Additional laser fluence therefore cannot substantially lower the SEY further.

### 2.4 Surface Analysis of LESS Processed Ferrite

To better understand the mechanisms behind this reduction in SEY the surface structures of the processed samples were further investigated. Observations using a SEM, see Figure 5, show that the Alpha series has significant regions of unprocessed material between the trenches. We see a sparse nanostructure (Figure 5d) made largely from material condensing out of the plasma cloud. This is contributing to its higher SEY. Within the trenches (Figure 5j) we see the dense, “feathery,” nanostructure we identified earlier as excellent for SEY reduction. There are no clear differences between the 8C11 and CMD 5005 samples, indicating that they are behaving similarly under ultrafast laser irradiation though this is not conclusive.

Images of the improved processing clearly show why the SEY was further reduced, as the entire surface is now constructed of trenches covered in a dense nanostructure. At the higher powers of the CMD samples, Figure 5k,l, the nanostructure loses it's ‘feathered’ appearance and becomes pockmarked instead. These are also high aspect ratio features that will drive SEY reduction. Which structure is more effective is not possible to determine as it is coupled to the microscale trench aspect ratio. At these higher fluences the trenches are less structurally consistent, with material bridging across trenches and more variation in peak shape. This has not been detrimental to SEY reduction but may reduce the consistency of other surface properties. This is especially pronounced in the CMD Cross parameters with some evidence of minor melt flow, l. This indicates the fluences used here are likely higher than necessary as the same level of SEY reduction was achieved at half the average power with the 8C11 cross parameters.

Analyzing the surface composition through EDX reveals that the process has caused significant changes to the elemental composition, see **Table** **4**. There is a significant drop in carbon as the laser irradiation has removed many of the hydrocarbon contaminants. The level of reduction correlates with increased exposure to the laser. There is a significantly increased presence of zinc. The cause of this is unclear, particularly as there is not a similar increase in the propensity of nickel. ZnO, as a very stable compound, may be preferentially formed at the high temperatures and pressures experienced either on the surface or in the plasma plume. We believe the reduction in iron is purely relative due to the increased presence of zinc and nickel. The constant level of oxygen would imply that the surface remains close to fully oxidised which is expected when processing in air.

**Table 4:** The surface composition of processed ferrites as determined by EDX. Values are the atomic percentage (at.%) of each element. The “Other” column denotes other elements present at trace amounts. Note the significant drop in carbon content and the large increase in zinc compared to unprocessed ferrite, Table 1.

	C	Fe	O	Zn	Ni	Other
8C11 Alpha	21.3	14.5	41.2	19.8	2.8	4.2
8C11 Linear	15.6	18.7	43.2	16.3	4.4	1.8
8C11 Cross	10.6	19.7	44.3	19.7	4.8	0.9
CMD Alpha	16.6	17.4	43.1	19.5	3.4	0.0
CMD Linear	10.0	14.6	43.9	27.1	3.5	0.9
CMD Cross	8.5	18.0	45.6	22.5	4.7	0.8

The exact proportion of SEY reduction attributable to surface chemistry rather than topographical changes is difficult to ascertain, as it requires many measurements of how surface chemistry and topography change with the SEY. Furthermore, changing the topography requires changing either the laser fluence or exposure level, both of which changes the surface chemistry, so the variables are entangled. Previous work on copper showed surface chemistry contributed between 10% and 30% of the SEY reduction depending on the laser fluence and hatch distance used.^[^ ^56^ ^]^ This was able to be isolated as the surface chemistry changes of copper are simpler than in ferrite. In this case our measurements indicate it is likely playing a more limited role. First, the SEY of ZnO is reported as approximately 1.6 while the SEY of NiO is not reported in the literature.^[^ ^81^, ^82^ ^]^ An increased prevalence of ZnO an NiO could only reduce the SEY to an average of their respective yields and then only if they formed 100% of the surface layers. However as the change in the relative prevalence by atomic percentage of Zn and Ni atoms only increases by between approximately 19 and 29 percentage points after processing this can only be a small factor. Much of the processed layer is still composed of Fe_2_O_3_, though possibly not in its polycrystaline form, and so is unlikely to have substantially affected the SEY. A reduction in hydrocarbon contaminants is another cause of surface chemistry derived reduction in SEY, assuming these contaminates raise the SEY as in copper.^[^ ^83^ ^]^ The carbon has only dropped by a maximum of 15 percentage points so again the effect is small. It is therefore clear that to have reduced the SEY below one, the reduction has largely been driven by the changes in topography.

The surface sheet resistance and specific resistivity of the samples was also measured before and after processing. Before processing the samples had surface resistance and resistivity higher than was measurable with the setup (see Section Resistivity Measurements) at ⩾2.0 × 10^10^ Ω and ⩾2.6 × 10^7^ Ω*m* respectively. LESS processing has dramatically reduced the surface electrical resistance by several orders of magnitude, see **Table** **5**. The exact cause of this is yet to be determined, although it is likely associated with the changes in surface composition, the topographical changes or both. It is clear the surface resistivity decreased more with the more aggressive processing of the Linear and Cross parameters. The shallower trenches of the Alpha parameters have resistivity an order of magnitude higher than the more aggressively processed samples. This shows it is possible to find a balance between resistivity and SEY reduction despite the mechanisms for the change in resistivity not yet being clear. Also notable is that CMD 5005 Linear and Cross has higher resistivity than 8C11 despite the higher processing fluence. This may be due to the pockmarked rather than feathered nanostructure, although a mechanism for this is not clear. This dramatic reduction in resistivity may cause issues with eddy currents which can lead to heating and will oppose the desired magnetic field, increasing rise times. Careful consideration will be needed to balance the SEY of the ferrite surface against this reduction in resistivity in any final application.

**Table 5:** Properties of the LESS treated ferrite. Average measurements of depth and aspect ratio are taken from cross sections processed ferrite across an average of 10 trenches. See Section 4 for details about other values.

	8C11	8C11	8C11	CMD	CMD	CMD
	Alpha	Linear	Cross	Alpha	Linear	Cross
SEY Maximum	1.27	1.14	0.81	1.20	0.81	0.81
PEE (eV)	700	500	1500	575	1500	1500
Surface Sheet Resistance (Ω)	7.4 × 10^6^	1.5 × 10^4^	8.8 × 10^4^	3.9 × 10^6^	6.2 × 10^4^	4.3 × 10^5^
Surface Resistivity (Ω m)	1.0 × 10^4^	2.1 × 10^1^	1.2 × 10^2^	5.4 × 10^3^	8.6 × 10^1^	5.9 × 10^2^
Trench Depth (µ m)	6.1 ± 0.7	15.8 ± 1.7	15.4 ± 2.6	6.0 ± 0.7	34.2 ± 2.6	23.3 ± 3.1
Aspect Ratio	0.65 ± 0.10	1.46 ± 0.10	1.39 ± 0.21	0.66 ± 0.06	2.78 ± 0.38	2.42 ± 0.36

All six LESS processing regimes were replicated on identical ferrite samples which were encased in resin and polished in order to reveal their cross sections, more details in Section Cross Sections and the supplementary document. Measurements of the trench depth and aspect ratio were taken and are presented in Table 5. The brittle nature of ferrite makes it difficult to produce a truly smooth surface. This leaves voids leading to significant error in the measurements. We can see however both Alpha samples exhibit trenches with an average depth of 6 µm and an aspect ratio, defined as the depth divided by the width of the top of the trench, of 0.65. This aspect ratio would not typically be expected to significantly lower the SEY on its own, showing the nanostructure and surface composition changes are the main causes of the lower SEY.

The 8C11 Linear and Cross parameters produce trenches 15 µm deep with an aspect ratio of 1.4. The reduced focal spot diameter has therefore had the desired effect. This aspect ratio is typically predicted to cause significant SEY reduction and the similarity of the nanostructure to the Alpha series supports this. The further reduction with the topography change to the Cross series also confirms that the micro topography is now a significant SEY reducing factor.

The CMD Linear parameters further deepen the trenches to 34 µm, improving the aspect ratio to 2.8. This is at or beyond the point to fully minimise the SEY of ferrite. Interestingly the cross hatch pattern reduces the depth and aspect ratio to 23 µm and 2.4 at this laser power. This may be due to the second pass of the laser causing minor melt flows to fill the bottom of the trenches. The high laser fluence of these parameter sets is likely more than necessary to reach minimal SEY with a linear trench pattern, with the ideal fluence for this laser spot size likely being between 0.4 and 0.6 J cm^−2^.

**Figure 6:**
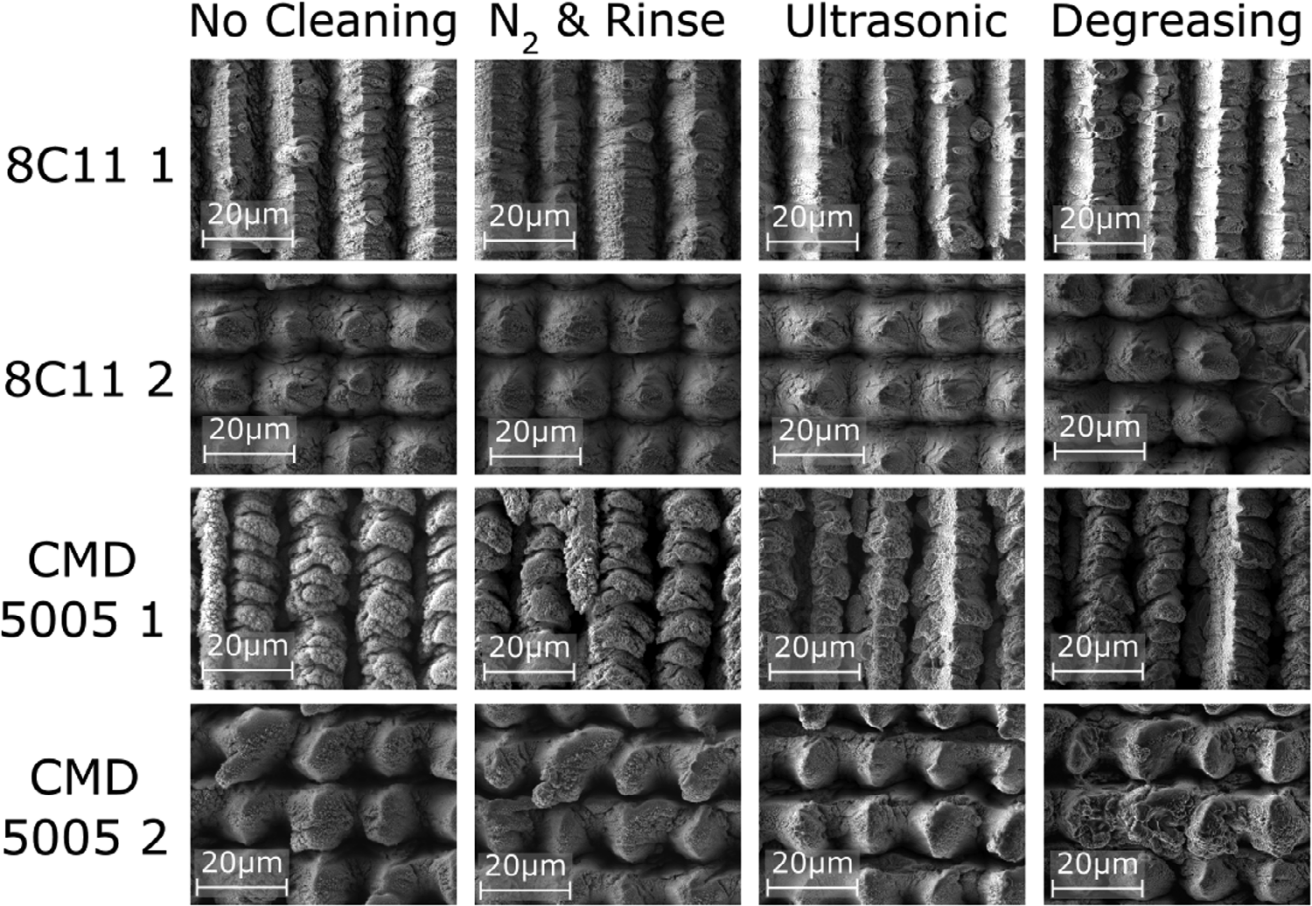
SEM images of the processed ferrite samples after different levels of cleaning. Images are at 2000x magnification.

Finally the higher fluence samples underwent three levels of cleaning. First, blowing with 2 bar *N*_2_ followed by rinsing in deionised water and isopropyl alcohol. Second, ultrasonic cleaning in deionised water followed by the first level of cleaning. Third, immersion in a degreasing agent followed by ultrasonic cleaning in the same degreasing agent and rinsing in deionised water, it is then baked at 60 °C for one hour, followed by the second and then first level of cleaning. Full details in Section Cleaning. These sample areas were then compared via SEM to an area that had undergone no cleaning in order to test the stability of the nanostructure, see **Figure** **6**. This cleaning regime is similar to that applied to copper at CERN to enure its applicability.^[^ ^84^ ^]^

Figure 6 shows that the more aggressive cleaning has removed more of the nanostructure from all the samples, particularly at the tops of the trenches. Deeper within the trenches the nanostructure appears largely undisturbed though it is difficult to image as the trenches are strong electron absorbers. Higher magnification images are in the supplementary document. Light cleaning with *N*_2_ gas and rinsing seems to have removed very little nanostructure. What is removed we suspect is largely poorly bonded particulates that condensed out of the plasma plume. It seems unlikely that this was a major contributor to SEY reduction. Untrasonication and degreasing removed more material particularly for the lower fluence 8C11 parameters. This will have increased the SEY though we believe this will be somewhat minor similar to observations in copper.^[^ ^84^ ^]^ More work is needed to balance the needs of vacuum compatibility with SEY reduction for application within particle accelerators.

## 3 Conclusion

Future work will be focused on scaling up this process to a full size magnet, mitigating the increase in surface conductivity and on investigating the effects of LESS processing and subsequent cleaning on the vacuum compatibility, beam coupling impedance, and magnetic properties of the ferrite. Scaling up the process will come with challenges on maintaining the consistency of the processing and finding ways to increase the processing speed. This can be addressed with parallel processing and changing the processing parameters, for example increasing the fluence to maintain the trench shape with an increased scan speed. Long term processing at 257 nm can cause issues with degradation of the optics. This can be avoided by using a longer wavelength. Processing a full magnet block will allow for the full measurement and comparison of the impedance and magnetic performance. We expect the surface structures to cause a minor increase in beam coupling impedance as was seen in copper,^[^ ^84^ ^]^ but this will likely be wholly offset by the large decrease in surface resistivity. We also expect a very minor effect on the magnetic properties as these are bulk properties and LESS has only modified the top 35 µm. Optimisation of the processing parameters can the be made to further mitigate any changes.

In summary, we have expanded the applicability of laser engineered surface structures for SEY reduction to a dielectric ceramic for the first time. Previously, the technique had only been performed on metals. Here we demonstrate how LESS methodology can be applied to a broad range of solid surfaces to reduce the SEY. LESS is a simple process consisting of removing material from the surface using laser ablation to generate SEY reducing micro and nanostructures. As these are topographical changes they are material independent, provided the material can be ablated by laser radiation. It can therefore be applied to other relevant technical materials in the fields of compact linear accelerators, radio frequency windows and satellites such as alumina, magnesium oxide and indium oxide.^[^ ^31^, ^45^, ^85^, ^86^ ^]^ Careful study of the material both before and after processing allows for optimisation of the lasing parameters in order to find the minimal SEY possible with the material using LESS. Alternatively other key parameters such as surface composition, trench depth or processing speed can be optimized in conjunction with an acceptable SEY reduction.

More specifically, we have applied LESS to Ni/Zn ferrite demonstrating a promising path to mitigate ECs inside the aperture of the kicker magnets operating in the particle accelerators at CERN. This work has, for the first time, optically characterized and thoroughly investigated the light matter interactions of Ni/Zn ferrites with ultrafast laser pulses. We have used these novel findings to reduce the SEY maximum of these ferrites from 2.0 to 0.81, the experimentally determined minimum possible using LESS. This is a crucial step toward preventing ECs from forming in the ferrite kicker magnet apertures, and preventing the instabilities that pose a significant limitation for long duration high intensity circulating beams as observed within the SPS.^[^ ^87^ ^]^

## 4 Experimental Section

### Electron Dispersive X‐Ray Spectroscopy

EDX measurements were performed using a ZEISS Sigma Field Emission Gun Scanning Electron Microscope equipped with InLens Secondary Electron, Everhart‐Thornley Secondary Electron, and back‐scattered electron detectors for imaging. A 50 mm^2^ X‐Max EDS detector and AzTEC software from Oxford Instruments were used for chemical analysis. The SEM‐EDS investigation was conducted between 5 and 10 keV with a 60 µm aperture and an acquisition time of 120 s for all specimens, over areas of approximately 100 by 25 µm.

### Optical Spectroscopy

The reflectivity and absorption of the ferrite samples was measured using a JASCO V670 spectrometer with an integrating sphere accessory. Measurements were normalized against both a dark measurement with the light source blocked and an alumina reference block representing 100% reflectivity across the spectrum. Measurements were taken between 1200 and 200 nm with a 1 nm data interval. The grating was changed at 850 nm, hence the drop in noise. A 8 nm slit window was used to mitigate the noise that was then reduced to 2 nm to improve the resolution in the visible and UV. The light source was changed from a halogen lamp to a deuterium lamp at 350 nm. The optical emissivity was measured on a Bruker vertex 70 spectrometer using an A562 integration sphere.

### Damage Threshold Measurement

The method outlined in Section 2.1 is only valid if the laser beam is approximately gaussian. These laser outputs were confirmed gaussian using a Spiricon SP620U CCD Camera. The values of ϕ_*th*_ in Table 2 are taken from the extrapolated *x* intercept of plotting Equation (3) and applying a linear polynomial fit. The errors were calculated as the 95% confidence intervals of that fit. Where trenches are inscribed, the trench width is substituted for the spot diameter as *D* in Equation (3). Observations of the DT spots and lines were made using a Keyence VHX‐1000 optical microscope with a variable magnification up to 5000x and integrated measurement software.

### SEY Measurement

All SEY measurements were conducted under ultra high vacuum of 5 x 10^−8^ Pa. The samples were negatively biased to –18 V and two Femto DHPCA‐100 current amplifiers are used to monitor the current from the sample and a collector biased to +45 V. The collector was shielded from external magnetic fields. As ferrite was an insulator it will accrue charge during electron bombardment. This charging could then deflect incoming electrons affecting the measurement of SEY. To avoid this a pulsed measurement technique was used where an ELG‐2/EGPS‐1022 electron gun emits 50 µs electron pulses containing a maximum charge of 2 x 10^−12^ C that impinge on the sample surface. Between pulses the sample underwent 5 eV continuous electron exposure for 2 min to alleviate any accrued charge from the sample and prevent the negative effects of charging. The sample and collector currents were continuously monitored to check for signs of charging and measurements were repeated for consistency. As the processed samples had higher conductivity, any risk of charging was expected to decrease after LESS processing. Several ∼1 mm^2^ areas of each sample were measured and an average taken of the results to account for local inhomogeneities. The energy of the electrons in pulses was varied from 150 eV through to 1500 eV to measure the SEY at all relevant PE energies. Further details can be found in ref. [88].

### Surface Roughness Measurements

Surface roughness was measured using a Dektak XT Stylus Profiler. A 5 mm long profile was measured with a lateral resolution of 0.5 µm and depth resolution of 8 nm. A levelling algorithm was applied across the profile and then the average roughness was calculated across the whole profile.

### Laser and Motion Control Setup

All experiments were carried out using a Light Conversion Carbide femtosecond pulsed laser. This was paired with a harmonic module giving access to 40W of 1030 nm light, 20W of 515 nm, and 4W of 257 nm. These output from three apertures after the harmonic module. One for each wavelength. The 1030 and 515 nm beam lines were combined through the use of a flip mirror and directed toward the sample with dual order Nd:YAG laser line mirrors, through a plano‐convex lens with dual anti‐reflection coating, mounted on the z‐stage. The 257 nm beam was directed in parallel to the other beam line with Nd:YAG 4th harmonic laser line mirrors. It goes through a separate UV fused silica AR coated lens on the z stage. In addition two further plano‐convex lenses were used before the z stage as a 4f‐telescope to increase the collimated beam size and reduce the focal spot size. The x, y, and z stages were all manufactured by Aerotech and managed by custom software designed in house that fully integrates the motion control with the shutter control of the laser, allowing for sub micrometer precision. Focal position was maintained through use of a distance sensor in conjunction with a custom built digital optical microscope mounted to the z stage. The samples were secured through custom spring loaded mounts.

### Scanning Electron Microscopy

The SEM images shown in Figures 3 and 6 were taken at Dundee using a JEOL JSM‐7400F field emission scanning electron microscope. The images in Figure 5 were taken at CERN using the same system as the EDX measurements using a 3 kV voltage.

### Resistivity Measurements

The resistivity of the samples were characterized using a 4‐point probe setup based on a linear array of four tungsten carbide needles with a 0.4 mm diameter and 100 µm tip radius. They were separated by 1 mm. The system was driven by an ISO‐Tech IPS‐303D laboratory DC power supply and the current and voltage were measured respectively by Keithley 6517B and PREMA DMM 6000 digital multimeters. The sheet resistance measurements assume a conductive layer on an insulating substrate (affirmed by the high resistivity before processing and the lack of modification below the trenches after processing as seen by the cross sections). Resistance was then calculated as $R_{□}=\frac{U}{I}\cdot \frac{\pi }{\ln(2)}$ and the resistivity as $\rho =\frac{U}{I}\cdot 2\pi \cdot s$. Where $R_{□}$ is the surface sheet resistance, *U* is the potential difference between the inner probes, I is the current through the outer probes and s is the tip spacing. For more details see.^[^ ^89^ ^]^

### Cross Sections

The cross sections of the processed ferrite were prepared on separate 8C11 and CMD 5005 samples to the SEY, SEM, and EDX measurements. All three parameter sets were processed onto the edge of the samples in 1 mm square areas. The samples were then encased in Lam Plan 603 mounting resin and then progressively polished down starting with 120 grit sandpaper and finishing with 0.25 µm grit diamond paste. The cross sections were then examined using the same Keyence VHX‐1000 microscope as for the DT measurements. Trench depths were measured by the average level of the two plateaus either side of the trench to the point at the bottom. Trench widths were calculated by extrapolating the bottom angle of the trenches with the measured trench height using the following equation: $w=2h\tan\left(\frac{\theta }{2}\right)$, where *w* is the trench width, *h* is the measured height and θ is the bottom angle. All values were averaged across 10 trenches and errors are the standard deviation.

### Cleaning

The 8C11 1, 8C11 2, CMD 5005 1, and CMD 5005 2 laser processing regimes were repeated on two additional samples. Each received a different level of cleaning. One received no cleaning. The next was blown with 2 bar nitrogen gas before rinsing in deionised water and isopropyl alcohol followed by further nitrogen blowing. The next sample underwent ultrasonic cleaning in deionised water for 10 min and then received the same treatment as the previous sample. The next sample was immersed in a degreasing agent for 1 h and 45 min before ultrasonic cleaning in the same degreasing agent for 15 min. It was then rinsed for five minutes in deionised water. It was then allowed to air dry and then baked at 60 °C. It then underwent the previous two cleaning procedures. A final sample was left untreated. These were then all examined under SEM at the University of Dundee.

## Conflict of Interest

The authors declare no conflict of interest.

## Supporting information

Supporting Information

## Data Availability

The data that support the findings of this study are openly available in Ferrites with a Minimised Secondary Electron Yield ‐ Data Ferrites with a Minimised Secondary Electron Yield ‐ Data at 10.15132/10000257, reference number 0.
