# Supplementary material for: Ferrites with a Minimized Secondary Electron Yield
Source: Adv Sci (Weinh). 2025 Jan 17;12(10):2410083. doi: 10.1002/advs.202410083 (PMC11905002; doi:10.1002/advs.202410083)
Supplement: Supplementary file 1 — Supporting Information [file ADVS-12-2410083-s001.pdf]

Supporting Information

for *Adv. Sci.*, DOI 10.1002/advs.202410083

Ferrites with a Minimized Secondary Electron Yield

Robin Uren, Manuel Hoffman, Amin Din, Stefan Wackerow, Holger Neupert, Stephan Pfeiffer, Alice Moros, Michael Barnes, Giorgia Favia, Marcel Himmerlich and Amin Abdolvand**

Supporting Information
Ferrites with a Minimised Secondary Electron Yield

Dr. Robin Uren[1*], Dr. Manuel Hoffman[1], Amin Din[1], Dr. Stefan Wackerow[1], Dr. Holger Neupert[2], Dr. Stephan Pfeiffer[2], Dr. Alice Moros[2], Dr. Michael Barnes[2], Dr. Giorgia Favia[2], Dr. Marcel Himmerlich[2], Prof. Amin Abdolvand[1**]
1 School of Science and Engineering, University of Dundee, Nethergate, Dundee, DD1 4HN, UK.
2 CERN, European Organisation for Nuclear Research, Meyrin, Geneva, 1211, Switzerland.
* ruren001@dundee.ac.uk, ** a.abdolvand@dundee.ac.uk

1 High magnification

Figure S1 shows the highest magnification images taken for each sample giving more detail of the nanostructure. All images are taken within the trenches. We see a change in structure as the fluence was increased from a more bubbly, feathered appears to a a more globular and pockmarked appearance. We also see a decrease in uniformity as the fluence increases. This is likely due to additional heating disturbing the formation of these structures. This may be a slightly weaker structure for reducing the SEY however this is hard to quantify. It is clear that if this is the case the increased micro-trench aspect ratio is more than compensating for this.

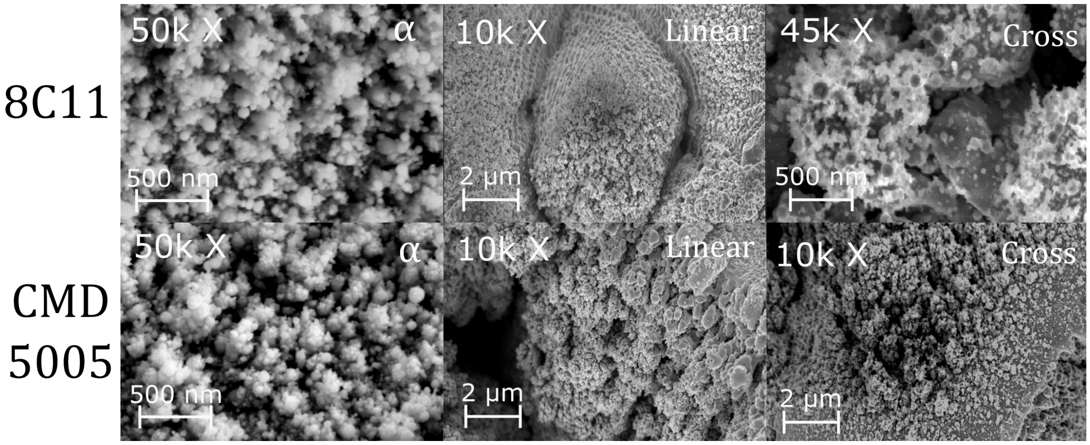

Figure S1: High magnification images of the processed ferrites. Top row is 8C11, bottom is CMD 5005. Images were taken at CERN with details given in in Section 4.6.

2 Cross Sectional Images

As ferrite is a brittle material it is challenging to produce high quality cross sections for measuring the trench depth. As such other methods including measuring the change in optical focal depth between the bottom and top of the trenches when viewing the surface, and using a Veeco optical profilometer were attempted. These however gave inaccurate results as it showed that increasing fluence did not increase the trench depth. We believe this is due to the nanostructure obscuring the true bottom of the depth, partly as the structures are on the scale of hundreds of nanometers so heavily scatter visible light. Images of the cross sections were taken at multiple magnifications, the highest presented below in Figure S2. We can see voids created by the polishing process which adds error to the measurements. Through careful optimisation of the polishing process and by encasing the samples in epoxy these voids were minimised allowing for useful trench depth and width. To preserve the integrity of the initial samples, the three process parameters were repeated on separate samples of CMD 5005 and 8C11 before being encased in resin and polished.

3 Cleaning Images

SEM images were taken of the cleaning samples with a focus on the nanostructure within the trenches, see Figure S3. We can see the removal of nanostructure is less severe within the trenches with the more aggressive cleaning techniques de-

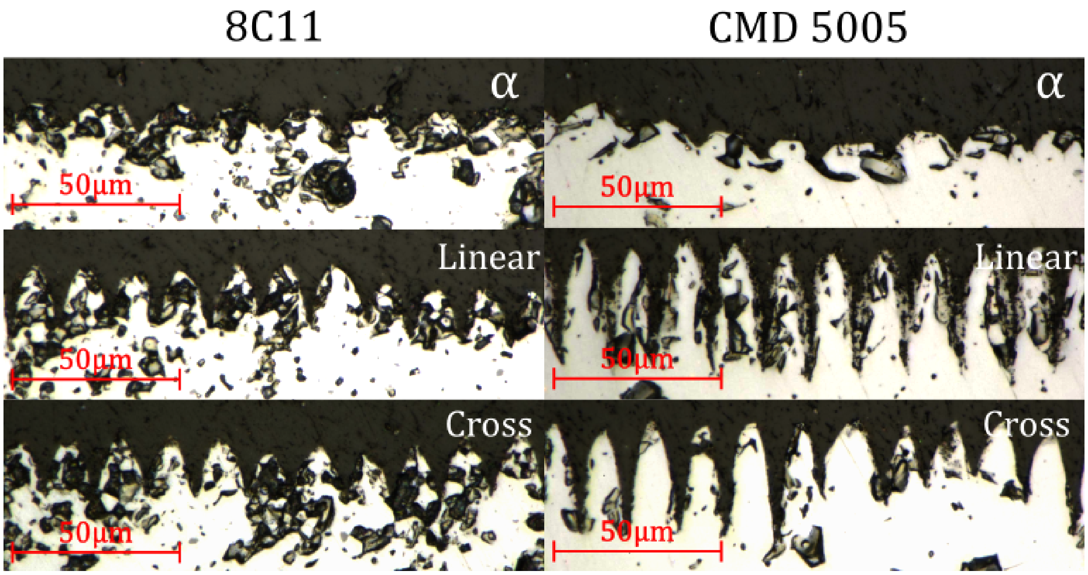

Figure S2: Cross sectional images of the processed ferrites. Details given in in Section 4.8.

scribed in section 4.10 of the main article. We also see the higher fluence processing seems to retain its nanostructure more successfully.

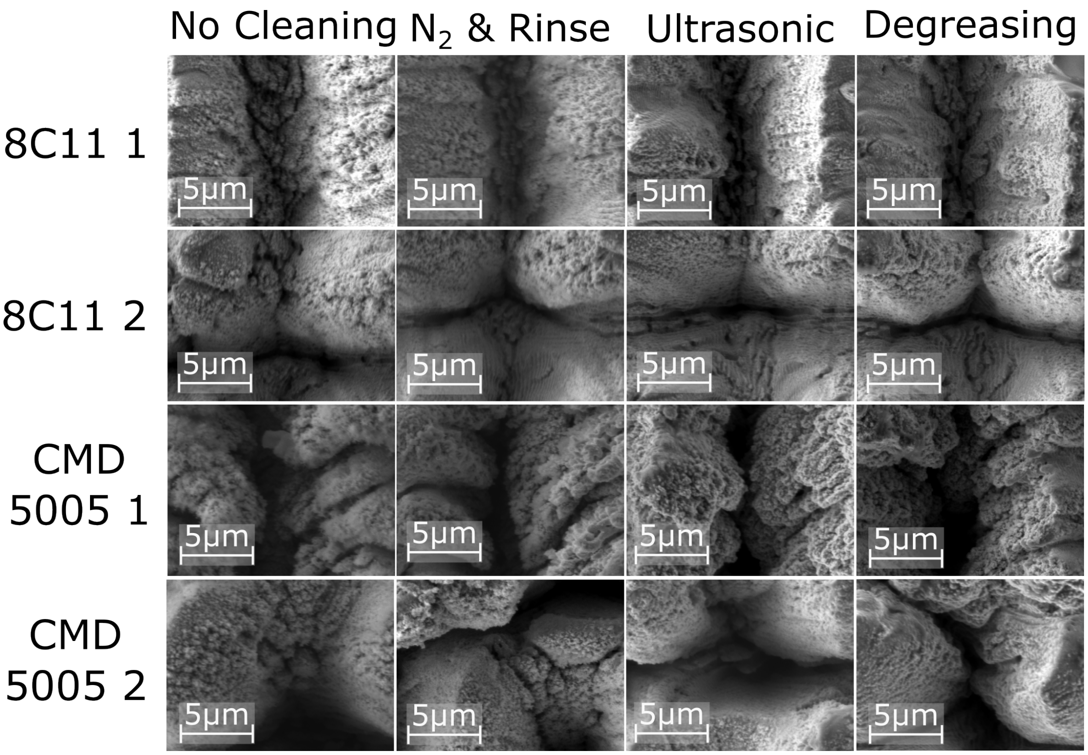

Figure S3: Higher magnification SEM images of the cleaning samples. All images are at 7500x magnification.
